# Amyloid fibril polymorphism: Structural mechanisms of assembly and the links to disease

**DOI:** 10.1016/j.sbi.2026.103245

**Published:** 2026-03-13

**Authors:** Alexander I. P. Taylor, Sheena E. Radford

**Affiliations:** Astbury Centre for Structural Molecular Biology, School of Molecular and Cellular Biology, Faculty of Biological Sciences, University of Leeds, LS2 9JT, UK

## Abstract

Amyloid fibrils are involved in devastating conditions such as Alzheimer’s disease, Parkinson’s disease, Huntington’s disease, and systemic amyloidosis. They exhibit polymorphism, meaning that a single protein sequence can adopt different amyloid folds that vary with time and self-assembly conditions. Polymorphism confounds structure-based drug design and raises fundamental questions regarding why particular fibril structures form and how they cause disease. Here, we highlight the latest advances in our understanding of amyloid polymorphism, including its structural basis, thermodynamic origins, kinetic influences, and significance for disease. The next frontier will be to predict fibril structures, disentangle the dynamic mechanisms that guide the progression of fibril polymorphs, and illuminate how cofactors and the physiological milieu select for particular polymorphs in disease.

## Introduction

Amyloid fibrils are ordered protein assemblies that are involved in widespread diseases, such as Alzheimer’s disease, Parkinson’s disease, Huntington’s disease, type-2 diabetes, prion diseases, and systemic amyloidoses [1]. They are formed by over 40 different disease-related proteins [2] and have diverse structures, but all share a common architecture termed the cross-β amyloid fold [1,3,4]. Treatments targeting amyloid fibril formation have shown promise for tackling these devastating conditions [5], but are hindered by amyloid polymorphism: the capacity for a single protein sequence to form amyloid fibrils with many different structures [3,6]. Polymorphism presents a challenge for structure-based drug design and raises fundamental questions regarding how amyloid fibril structures form and the relationship between structure and disease presentation.

Recently, cryogenic electron microscopy (cryo-EM) has fueled an explosion in the number of amyloid structures solved to near-atomic resolution [1,3,7], making it now possible to analyze large datasets of fibril structures to make sense of this diversity and uncover its underlying thermodynamic origins [8–11]. In addition, recent mechanistic studies have shed light on how proteins navigate the complex energy landscapes underpinning fibril polymorphism [12,13], and how changes in conditions or interactions with other molecules can steer polymorphism down a different path [13,14]. As a result, it is now possible to identify disease-related amyloid structural motifs that are promising therapeutic targets [7,15], and we are beginning to understand the processes that give rise to different fibril structures and how this relates to disease presentation [7,12,13,16].

Here we summarize the current understanding of amyloid polymorphism, including how polymorphs are generated and selected for *in vivo* and *in vitro*. We also discuss the mechanisms by which physiological conditions or cofactors funnel or steer polymorphism toward different fibril species, and how we can exploit this knowledge for future therapeutic interventions.

## The amyloid fold

**Figure 1:**
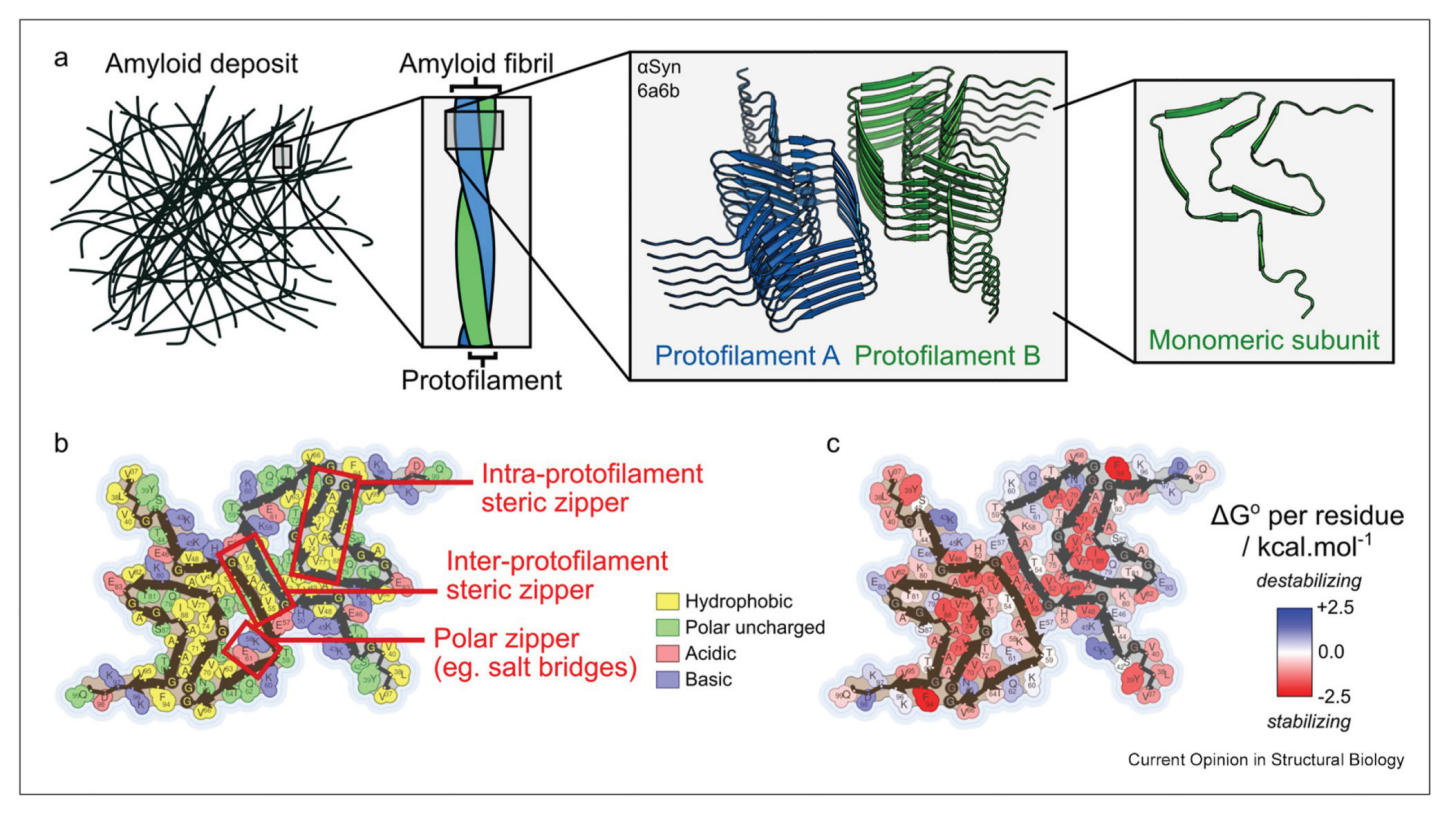
Key characteristics of the amyloid fold. **(a)** Amyloid deposits consist of amyloid fibrils, each of which in turn consists of smaller filamentous units called protofilaments. Each protofilament is a stack of typically thousands of monomeric subunits, as illustrated here with a six-layer segment of an α-synuclein (αSyn) fibril structure (PDB ID: 6a6b [63]). Monomeric subunits usually have a flattened structure and each β-strand is part of a different intermolecular β-sheet. **(b)** Hydrophobic amino acids are usually buried in the fibril core, whereas polar amino acids are usually exposed. Key stabilizing interactions include steric zippers formed where side chains interlock to optimize hydrophobic contacts, and polar zippers formed by salt bridges or hydrogen bonding. **(c)** Different residues may make either a favorable or unfavorable contribution to the free energy of the fibril, and stabilizing residues are usually located in crucial zippers in the core. Plots in panels **b** and **c** were generated for the same fibril structure (PDB ID: 6a6b [63]) using the Amyloid Atlas Illustrator Server [3].

Amyloid fibrils are characterized by their cross-β structure, named after their signature X-ray diffraction pattern with orthogonal reflections at 4.7—4.8 Å and 8—11 Å [17,18]. Structurally, this results from the presence of continuous intermolecular β-sheets that run along the fibril axis, with a single amyloid fibril typically consisting of thousands of stacked subunit layers that collectively form these β-sheets (Figure 1a) [1,3]. The length of amyloid fibrils can vary enormously, from hundreds of nanometers to tens of micrometers, corresponding to hundreds to many thousands of subunits [1,3]. However, simulations suggest that short fragments may be stable down to stacks of just five subunits [19], and diffusible fibril fragments are an increasingly recognized form of oligomer that has been detected in patient-derived samples [20]. Fibrils are hierarchically organized [16], with monomers stacking to form protofilaments that each contain one or more continuous intermolecular β-sheets, and which laterally associate with one another to form a complete fibril [1]. Typically, each constituent subunit has a flattened structure, optimizing its ability to hydrogen bond to the subunits above and below in the stack, and allowing each of its β-strands to be incorporated into a different ribbon-like intermolecular β-sheet (Figure 1a) [4]. However, some amyloid protofilaments, particularly those of functional amyloid, have layers that comprise different, complementary sequences [1,4]. The β-strands in a cross-β structure are usually oriented parallel and in-register with one another (Figure 1a) [1].

The extensive intermolecular β-sheets in amyloid fibrils entail the formation of massive, repetitive hydrogen bonding networks that run along the fibril axis [4]. As a result, hydrogen bonding has been suggested to make a greater contribution to the stability of amyloid fibrils than it does in globular proteins [21]. In agreement with this, density functional theory (DFT) calculations have shown that interactions between repetitively stacked backbone or side chain hydrogen bonding moieties induce a cooperative polarization of their electron density [22]. This gives amyloid fibrils unusually strong hydrogen bonds, with more favorable formation energies than those seen between water molecules in ice [22]. At the same time, complementary interactions support β-sheet hydrogen bonding to further stabilize the fibril structure. These additional interactions are ubiquitous in amyloid fibril structures, and are believed to be key stabilizers of amyloid formation as well as determining specific amyloid folds [1,3,4]. Stacking of monomeric subunits is also supported by side chain—side chain interactions, particularly π-stacking and amide side chain hydrogen bonding, and disfavored by unfavorable electrostatics that result from alignment of like charges. Orthogonal to the fibril axis, subunit folding and association of protofilaments are driven by the hydrophobic effect and/or formation of salt bridges between oppositely charged residues (Figure 1b,c). Due to the repetitive structure of amyloid fibrils, these supporting interactions occur in zipper-like arrays, most commonly “steric zippers”, in which the side chains on adjacent stacks of polypeptide chains form corrugated, often tightly mated interfaces that optimize van der Waals contacts, and “polar zippers”, in which adjacent stacks of polar moieties form salt bridges or hydrogen bonds with one another (Figure 1b) [3,4,23]. Due to the cooperativity between subunit stacking and folding, these complementary interactions all contribute to determining fibril structure. Non-protein cofactors may also be incorporated into the fibril structure and can play an important stabilizing role [6]. Lastly, the ordered cross-β core usually does not include the entire protein sequence, and many amyloid fibrils have a “fuzzy coat” consisting of disordered flanking regions than can span tens to hundreds of residues in length [24].

## Amyloid polymorphism: one sequence, many structures

**Figure 2:**
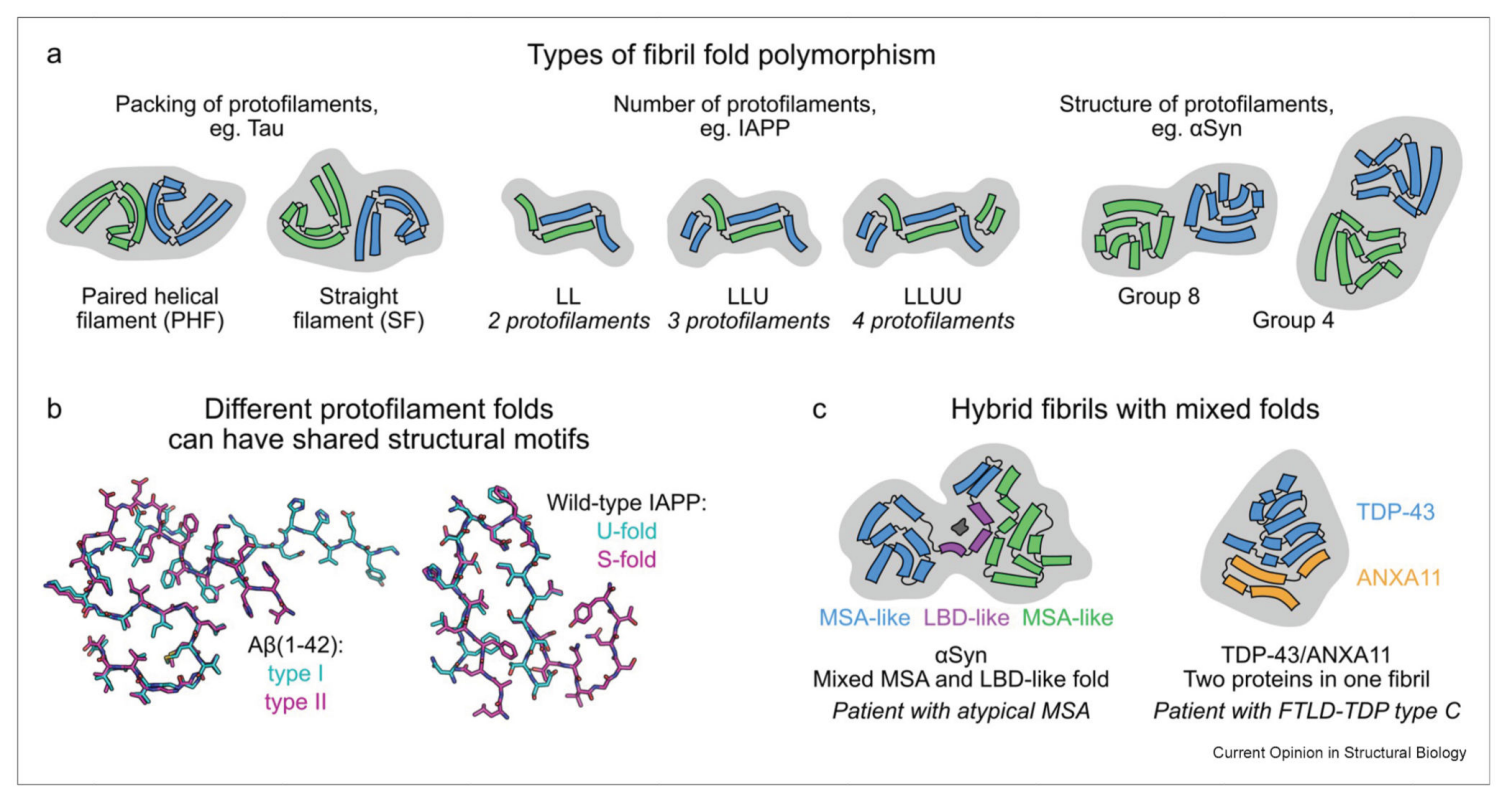
Types of structural polymorphism. **(a)** Fibril structural polymorphism can involve differences in packing interactions (e.g. PHF and SF of Tau [37]), number (e.g. IAPP [14]), or structure (e.g. αSyn, classified in Ref. [9]) of the constituent protofilaments or a combination of all three of them. As shown in the center, different protofilament structures may also occur in the same fibril (L-fold vs U-fold of IAPP). **(b)** Protofilaments with different structures can have shared structural motifs, exemplified by a similar S-shaped fold of many Aβ(1–42) fibril polymorphs [26] and a shared U-shaped motif in some IAPP fibril polymorphs [14], highlighted in the overlays. **(c)** The ability to combine different protofilament structures or structural elements can result in hybrid fibrils. For example, αSyn fibrils in a patient with atypical multiple system atrophy (MSA) were found to contain a substructural motif similar to that seen in Lewy body disease (LBD) [34]. Furthermore, a single fibril may contain protofilaments from two proteins with completely different sequences, such as TDP-43 and ANXA11 in a patient with frontotemporal lobar degeneration (FTLD) [64]. In panels **a** and **c**, the schematics are simplified views of subunits looking down the fibril axis, with curved bars representing β-strands and connecting lines representing turns or loops. The coloring of chains distinguishes between adjacent monomer subunits, and the light gray background serves to enclose protofilaments that together make up the fibril. In the hybrid αSyn fibril in panel **c**, the purple chain segment marks a substructure within the MSA-like protofilament that resembles that found in LBD and the dark gray shape is non-proteinaceous density. IAPP, islet amyloid polypeptide; PHF, paired helical filament; SF, straight filament.

In contrast with globular protein folding, a single protein sequence can usually adopt many different structures in the amyloid state, a phenomenon termed polymorphism [6]. Polymorphism can involve differences at every level of a fibril’s structural hierarchy (Figure 2a—c) [6]. Polymorphism exists for all types of amyloid fibrils, whether pathological, functional, or *in vitro* assembled.

For some disease-related proteins such as Tau or α-synuclein (αSyn), the amyloid fold observed across different individuals appears to be disease-specific [7,25], with different fibril structures associated with specific clinical presentations. However, this is not always the case as other disease-related proteins such as amyloid-β (Aβ) [26–28] and transthyretin [29,30] can form different fibril structures in the same disease, patient, or tissue. The diversity of antibody light chain (LC) sequences also means that different individuals with LC amyloidosis have different amyloid fibril structures, yet the same fibril structure has been found in different tissue deposits in a single individual [31,32]. Amyloid fibrils assembled from an individual protein sequence *in vitro* commonly result in an array of different structures under different but often closely related solution conditions (e.g. Refs. [7,12,14,33]), with multiple structures sometimes observed under the same conditions or even in a single self-assembly reaction [9]. This suggests that polypeptides have an intrinsic capacity to form different fibril structures, with specific features of the physiological environment selecting for a particular amyloid fold. Hence, cofactors such as metal ions, lipids, nucleic acids, or other ligands that associate with fibril structures (Figure 2c [34]), or the physicochemical environment of assembly (e.g. pH, cell type, subcellular location), can alter the structure that results. In turn, changes in fibril polymorphism may have important implications for disease. One possible explanation for disease-specific polymorphism is that different structures drive different pathologies. For example, αSyn fibril structures induced by binding heparin or covalent modification (e.g. phosphorylation or *O*-GlcNAcylation) have been shown to have reduced toxicity compared to fibrils formed under similar conditions without these cofactors or modifications [35,36].

## Different interacting regions yield an alphabet of amyloid folds

Polymorphism can involve different packing arrangements of the same protofilament fold (e.g. paired helical filaments (PHFs) and straight filaments (SFs) of Tau [37]), different numbers of protofilaments with a similar fold (e.g. β_2_-microglobulin [38]), or protofilaments with the same sequence adopting different folds (e.g. αSyn [9]) (Figure 2a). In the case of islet amyloid polypeptide (IAPP), polymorphism involves different numbers and combinations of distinct protofilament folds, resulting in an alphabet-like classification of structures (Figure 2a) [12,14,39]. Protofilaments with different folds may share common structural units, such as identical β-arch motifs in the S-type and U-type protofilament folds of wild-type IAPP [14], or an S-shaped kernel at the core of many Aβ(1—42) protofilament structures that otherwise differ in conformation (Figure 2b) [26]. These shared motifs may point to a common mechanistic origin, or the involvement of the same stabilizing cofactors or physicochemical influences on the generation of each fibril type. How this occurs, however, remains unknown.

Now that >700 amyloid structures have been solved to near-atomic resolution [3], the structures of amyloid fibril polymorphs can be classified into distinct topological groups, reminiscent of CATH and SCOP for globular proteins [40,41]. The approaches used have involved hierarchical [8,9,11] or density-based [10] clustering performed on various measures of the similarity of protofilament structures. In one such study, Connor et al. [9] analyzed 169 high-resolution structures of αSyn and Tau fibrils to produce hierarchical trees of fibril folds for both proteins. Similar structural classes were identified for a smaller set of Tau fibrils by Mullapudi et al. [8] and αSyn fibril core segments by Milchberg et al. [10], despite each study using different methods. This demonstrates that fibril polymorphs can be organized into well-defined, meaningful fold families based on similarity at the sub-protofilament level. Intriguingly, polymorphs formed under different conditions or by different mutants can fall in the same fold family, while others form distinct structures in response to seemingly minor changes in solution conditions. Hence, the determinants of polymorphism will be difficult to unpick. The existence of protofilament fold families also supports the notion that protofilament structures are built hierarchically by combining a restricted range of structural elements, whose capacity to form is apparently preprogrammed by sequence and solution conditions [12,42].

**Figure 3:**
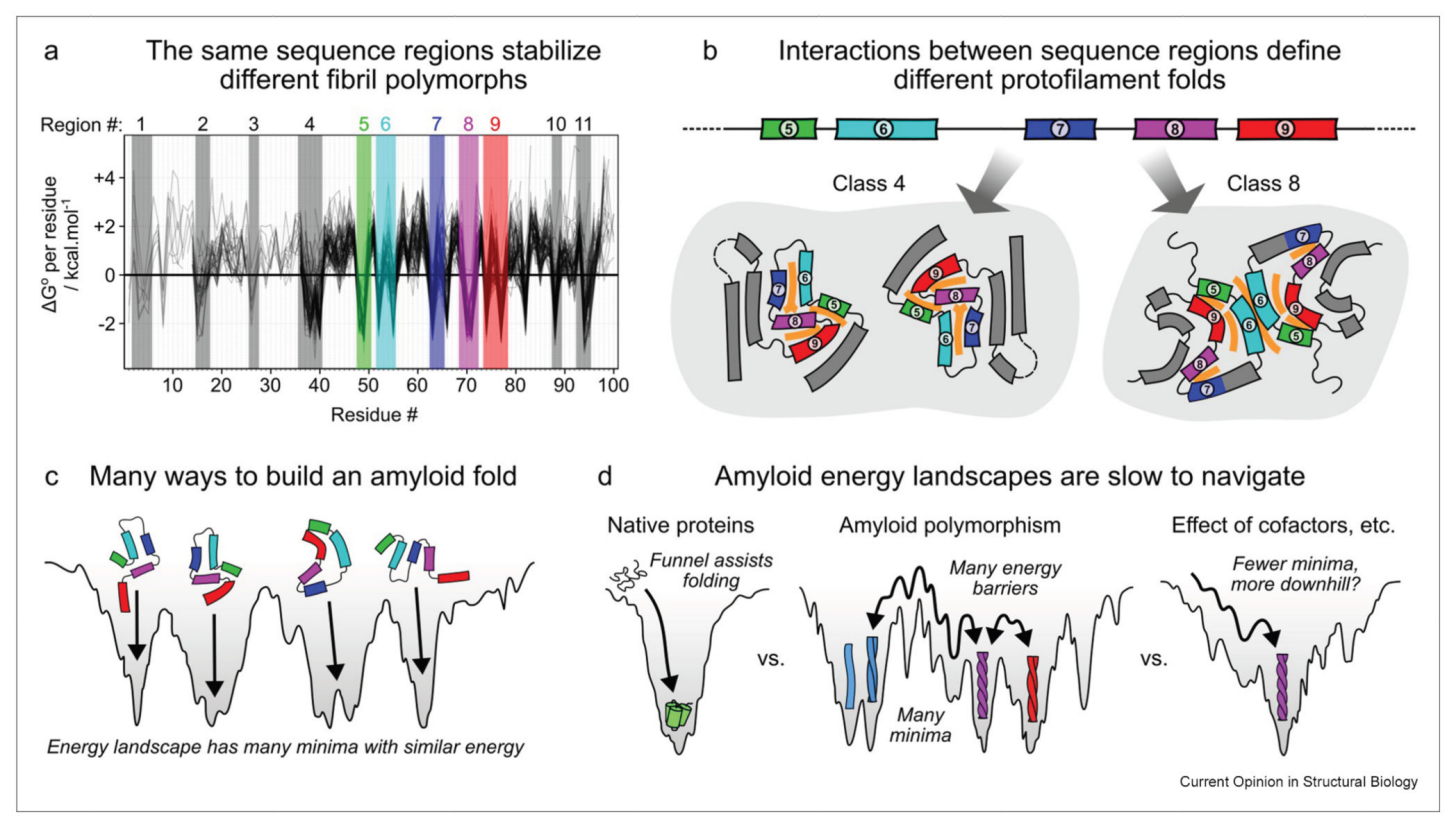
Interacting sequence regions drive polymorphic amyloid formation. **(a)** Overlay of the per-residue free energy contributions of individual residues in 100 different αSyn fibril polymorphs (gray lines), adapted from Connor et al. [9], showing that the same 11 regions stabilize different amyloid folds. Note that different residues form the fibril core in different structures, with residues 1–100 incorporated into the core in at least one structure. The C-terminal residues 101–140 (not shown) are never incorporated into the core. Regions 5–9 are relevant to the next panel and have been colored. **(b)** Different αSyn fibril structures have distinct and characteristic patterns of interaction between stabilizing sequence regions, as demonstrated with αSyn classes 4 and 8 from Connor et al. [9]. The schematics are simplified views of subunits looking down the fibril axis, with curved bars representing β-strands and connecting lines representing turns, loops, or disordered regions. Particularly discriminatory sequence regions 5–9 have been colored using the same scheme as panel **a**, while the rest of the sequence is colored gray. The light gray background encloses protofilaments that together make up the fibril. Orange bands between sequence regions reflect close intra- and inter-protofilament interactions, typically steric zippers. Note that the large gap among protofilaments in class 4 is due to the presence of a stabilizing ionic polar zipper, rather than a close steric zipper interface. **(c)** Different patterns of interaction can be used to build fibril cores with different topologies but similar stability, resulting in polymorphism. The schematics shown are representative of four of the αSyn fibril classes (3, 4, 8, and 10, from left to right) identified in Connor et al. [9], and use the same coloring and representation of secondary structure as panel **b**. For clarity, we show only a single protofilament and focus on the part of the polypeptide chain containing sequence regions 5–9. However, fibrils in these classes often have more than one protofilament, which adds a further layer of complexity to the energy landscape of assembly. **(d)** In contrast to globular protein folding, the energy landscapes of amyloid fibrils can have many minima and energy barriers that impede conformational exploration [47,48]. However, cofactors or other aspects of the physiological milieu may stabilize particular polymorphs, reducing the number of minima and possibly having an effect similar to funneling. In this panel, the color scheme encodes different conformations rather than stabilizing sequence regions: black, monomer; green, an example globular fold; red, blue, and purple, various amyloid fibril polymorphs.

Defining the sequence regions that drive amyloid formation may hold the key to understanding how different protofilament folds are constructed. Although most polypeptide chains have an inherent capacity to form amyloid fibrils, some sequences are particularly amyloidogenic. Most proteins contain sequence regions that drive aggregation, termed aggregation-prone regions (APRs [43]), which have diverse sequences but the common properties of high β-sheet propensity, enrichment in aromatic, amide and hydrophobic sidechains, and high compatibility with steric zipper formation [44–46]. They are usually incorporated into the fibril core but a single APR may adopt diverse structures within the cores of different fibril polymorphs [12,13]. In parallel with this, Mullapudi et al. [8] and Connor et al. [9] demonstrated the existence of well-defined stabilizing regions in the sequences of Tau and αSyn, which typically make a favorable contribution to the free energy of the fibril across different polymorphs (Figure 3a). Some, but not all, of these overlap with known APRs. Interestingly, both studies observed distinct pairings (typically heterotypic steric zippers) between stabilizing regions in different fibril polymorphs. As a result, different fold families of Tau, αSyn, and likely other amyloid fibrils have characteristic, distinct patterns of interactions among the stabilizing regions within their cores (Figure 3b).

The inherent promiscuity of the interactions that support amyloid fibril structures presents a wide range of ways to build energetically similar fibril folds, leading to polymorphism (Figure 3c). This promiscuity may result from the unique organization of amyloid fibrils compared to globular proteins. Given the flattened structure of fibril subunits, which restrict folding to an approximately two-dimensional search for contacts, it is easy to see how relatively small deviations of the peptide backbone could allow a single stabilizing region to pair up with a wide range of other sequences (Figure 3b,c). In contrast, in the hydrophobic cores of globular proteins, where interactions must be optimized in three dimensions, such deviations may not be so easily tolerated.

## Energy landscapes of amyloid polymorphs

Promiscuous interactions among stabilizing regions suggest how a single protein sequence can form many different amyloid polymorphs. For example, following the model of Connor et al. [9], where up to *n* stabilizing regions (dependent on sequence) may be incorporated into a given fibril fold and each region may interact with any other stabilizing region, the number of achievable fold topologies is $\sum_{k=1}^{n}(\begin{array}{c} n\\ k \end{array})2^{k(k-1)/2}$, where *k* is the number of regions incorporated and $\left(\begin{array}{c} n\\ k \end{array}\right)$ is a binomial coefficient reflecting the choice of which regions to incorporate. This is combinatorially explosive: while five interacting regions would permit 1449 topologies, 10 would permit approximately 3.6 × 10^13^. Restricting the number of pairings allowed between regions―a plausible constraint to compare only isoenergetic polymorphs―limits the number of options to $\sum_{k=1}^{n}(\begin{array}{c} n\\ k \end{array})(\begin{array}{l} k(k-1)/2\\ r \end{array})$, where *r* is the specified number of pairings. However, even here the numbers can still be enormous. Undoubtedly, steric constraints will limit these options, but other forms of polymorphism not accounted for by this model could also add to the diversity. The overall message is the same: the number of potentially energetically similar fibril polymorphs is huge. It is an amazing feat, therefore, that a single polymorph is commonly found in individuals associated with a particular disease (at least as visualized using high-resolution cryo-EM) [7].

The enormous number of possible structures and the large distance between their energy minima in conformational space suggests that the energy landscape of amyloid assembly is complex, highly rugged, and difficult to navigate [47,48] (Figure 3c—d). An analogy has been noted between the energy landscapes of amyloid polymorphism and those of glasses [48], whose multitude of comparable minima results in long-lasting, kinetically trapped out-of-equilibrium states, with extremely slow dynamics of maturation and relaxation to equilibrium. In the absence of evolved funneling or biasing cofactors, the same may be true for amyloid fibrils, explaining why *in vitro* self-assembly reactions are often so polymorphic (Figure 3d). On the other hand, the presence of dominant polymorphs in many diseases suggests that the physiological environment is sufficient either to funnel the energy landscape to a global minimum, or at least to create a deep enough local minimum that formation of other polymorphs is restricted (Figure 3d). As discussed later, it is also possible that disease structures may simply reflect out-of-equilibrium polymorphs that nucleate first and then self-propagate via secondary nucleation and elongation, although this is neither proven nor guaranteed given the long timescales of *in vivo* assembly.

## Amyloid polymorphism can change over time

**Figure 4:**
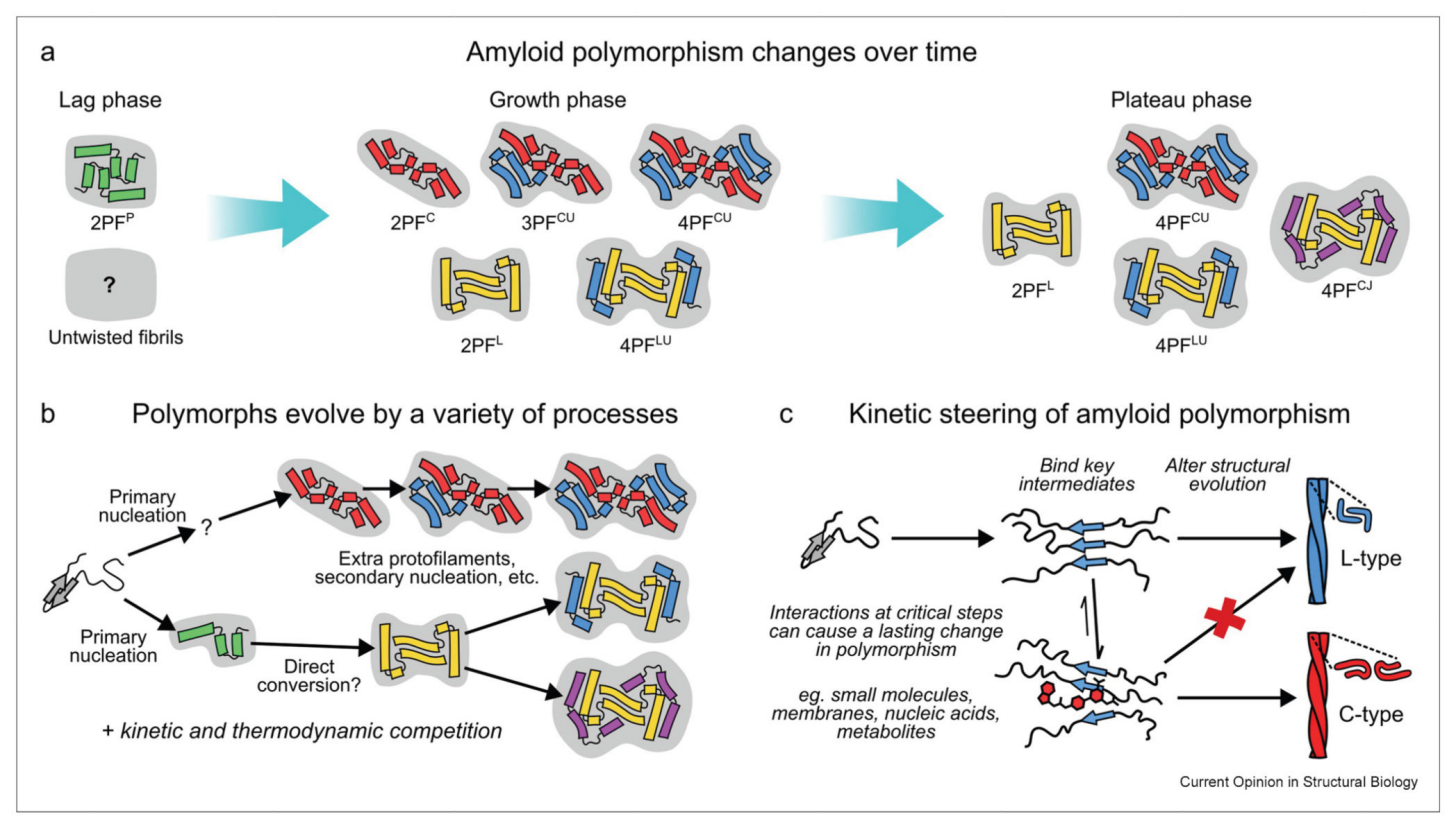
Kinetic effects in amyloid fibril polymorphism. **(a)** Amyloid polymorphism changes over time, as shown *in vitro* at high resolution by Wilkinson et al. [12] for the disease-related S20G variant of human IAPP. **(b)** A hypothetical reaction scheme showing the mechanisms by which amyloid polymorphism may change over time, using IAPP S20G as an example [12]. **(c)** Interactions with ligands or surfaces during critical steps that determine the evolution of fibril polymorphism can change the structural outcome; illustrated with the example of wild-type IAPP, where compounds that interfere with primary nucleation led to new fibril structures [14]. In panels **a** and **b**, the schematics are simplified views of subunits looking down the fibril axis, with curved bars representing β-strands and connecting lines representing turns or loops. The coloring distinguishes chains with particular subunit folds, with a single color corresponding to a single or small number of related folds. The light gray background serves to enclose protofilaments that together make up the fibril. For panel **c**, the color scheme corresponds to the lineage of fibril structure: gray, undifferentiated monomer; blue, L-lineage; and red, C-lineage as well as a small molecule (YX-I-1) capable of kinetically steering fibrils toward that lineage. IAPP, islet amyloid polypeptide.

The complexity of amyloid assembly energy landscapes means that it takes time for proteins to fully navigate them (Figure 3d), and polymorphism can depend on both the time of observation and the mechanism by which fibrils form (Figure 4). Early solid-state nuclear magnetic resonance (NMR) spectroscopy investigations showed that Aβ(1—40) forms a metastable amyloid polymorph under quiescent conditions *in vitro* that is replaced by a more stable polymorph later in the plateau phase [49]. More recently, other studies have demonstrated time-dependent changes in amyloid fibril polymorphism. For example, Pálmadottir et al. [50] showed that αSyn can form two different fibril polymorphs under identical conditions *in vitro*, with distinct morphology, structural properties, and interactions, but the less stable polymorph was gradually replaced by the more stable one as time progressed. In addition, Farrell et al. [51] demonstrated the occurrence of two different IAPP fibril polymorphs during self-assembly, with an initial polymorph formed by primary nucleation and a later one by secondary nucleation.

High-resolution structural insights into the time-dependence of fibril polymorphism have been provided by two recent cryo-EM studies. Wilkinson et al. [12] used time-resolved cryo-EM to track the self-assembly of IAPP S20G, a disease-associated variant that is more aggregation-prone than wild-type IAPP and forms polymorphic fibrils (Figure 4a). Remarkably, cryo-EM revealed small amounts of fibrils in the lag phase, most of which are untwisted (and hence unsuitable for structure determination using helical reconstruction) but 32 % of which are twisted, enabling their two-protofilament P-fold to be determined. In the growth phase, twisted fibrils become more abundant and distinct L- and C-lineages of fibril folds emerge. The L- and C-lineages exhibit further polymorphism resulting from differences in the number of peripheral protofilaments, with the average number of protofilaments increasing over time as the plateau phase is reached (Figure 4a). In spite of these gross changes in fibril structure, the energetic refinements are modest, mainly resulting from the addition of inter-protofilament interactions in the large fibrils that dominate in the late stages of assembly. Similarly, Lövestam et al. [13] tracked the progression of Tau fibril polymorphism in buffers that ultimately lead to either the Alzheimer’s disease (AD) or chronic traumatic encephalopathy (CTE) folds. They observed a similar increase in apparent twist and fold complexity over time, as well as a shared intermediate that occurred as the first cross-β containing structure under both conditions, and which they termed the first intermediate amyloid (FIA). Due to its order of occurrence, this was interpreted as the initial fibril polymorph formed by primary nucleation, prior to further structural maturation that leads to the AD and CTE folds.

While direct structural evidence for evolution of fibril structure *in vivo* has not yet been obtained, Fan et al. [52] recently demonstrated that αSyn fibrils seeded with cerebrospinal fluid from patients at different stages of Parkinson’s disease have different structures and pathogenicity depending on the disease stage, with more advanced disease showing enhanced seeding activity and induction of pathology in primary neurons. This suggests that a similar slow maturation of fibril folds may occur *in vivo* and contribute to disease progression.

## Polymorphism: kinetic or thermodynamic control?

The observation that amyloid polymorphism changes over time raises two key questions: how do new amyloid fibril polymorphs form during a self-assembly process, and how do kinetic and thermodynamic competition govern their changing abundance over time?

How polymorphs form and reorganize during assembly remains unclear. A variety of mechanisms must be involved, including primary nucleation, secondary nucleation on the surface of pre-existing fibrils, direct conformational conversion among early polymorphs that retain sufficient structural flexibility, and errors in templating during elongation (Figure 4b) [12,13,51,53]. Once formed, these polymorphs compete kinetically for monomers for growth and the fastest to self-replicate by elongation and secondary nucleation (or fragmentation) may then dominate [16]. In the plateau phase, when the monomer concentration approaches the solubility limit, kinetic competition is expected to give way to thermodynamic competition. Although very stable, amyloid fibrils exist in a slow dynamic equilibrium with monomers in solution [49,54,55] and undergo net disassembly if monomer levels decrease to below a certain critical concentration [54,55]. As monomer concentrations decline progressively throughout the plateau phase (at least *in vitro*), metastable polymorphs will begin to disassemble, starting with those that are least stable. As the least stable fibrils disassemble, the monomers released are repartitioned into more stable fibrils, until all monomers have been incorporated into the most stable fibril polymorph and a true monomer—fibril equilibrium can be reached [55]. However, we note that the situation *in vivo* could be very different, with continuous production of monomers by protein synthesis allowing more than one polymorph to remain stable indefinitely.

As well as thermodynamic stability, active processes may exert further selection pressures *in vivo*. Patient-derived amyloid fibrils formed by Aβ, serum amyloid A, antibody LC, and transthyretin exhibit enhanced protease resistance compared to fibrils formed *in vitro* by the same proteins, suggesting that only the most protease-resistant strains persist *in vivo* [56,57]. Differences in amyloid core structures and exposed fuzzy coats may also affect disaggregation by chaperones, as has been recently shown for different fibril polymorphs of αSyn [58]. Thus, the polymorphs that prevail in human disease may depend on a combination of strict thermodynamic stability and resistance to physiological quality control mechanisms. Nonetheless, an equivalent measure of effective stability is likely to govern competition between fibrils and drive changes in polymorphism.

Kinetics can also leave a lasting imprint on fibril polymorphism that persists deep into the plateau phase. Recently, we showed that two inhibitors (YX-I-1 and canagliflozin) profoundly affect the fibril polymorphs of IAPP formed at the end of fibril growth [14]. Neither compound achieves this simply by stabilizing an alternative fibril polymorph, but instead by interfering in early steps that determine fibril structure (Figure 4c). We thus propose the term *kinetic steering* to describe a lasting change in fibril polymorphism that is wholly or partly kinetic in origin, rather than simply involving differential stabilization. Crucially, the fact that a change in polymorphism can last deep into the plateau phase suggests that the fibril structures that form without inhibitors are either not present or unable to thermodynamically out-compete the altered polymorphs. In support of this, we found that fibrils of wild-type IAPP with 2PF^S^ and L-family structures [14,39], which were dominant in the absence of an inhibitor, were undetectable in kinetically steered samples, suggesting that their formation is strongly suppressed [14]. Thus, kinetic modulators can exert a long-lasting effect on polymorphism by filtering the “palette” of polymorphs that initially forms, and on which thermodynamic selection can then operate. In principle, kinetic steering could also operate *in vivo*, and our results show that even weak, transient interactions between small molecules and proteins can lead to a sustained interaction as aggregation progresses, ultimately leading to a profound effect on fibril structure [14]. This suggests that interactions with drugs, metabolites, or other biomolecules present in the physiological milieu could profoundly affect the kinetic evolution and structure of amyloid fibrils observed.

## Summary and outlook

The recent explosion of high-resolution amyloid structures, advances in computational approaches, and accumulation of mechanistic data have led to major advances in our understanding of fibril polymorphism. Despite this progress, fundamental questions remain.

Firstly, the rules that govern the pairing of sequence regions and selection of a particular amyloid fold are not understood. Addressing this gap will require new kinetic and structural analyses mapping how sequence regions pair to build fibril folds, as well as targeted experiments that seek to alter fold topology by mutagenesis, sequence modification, or addition of cofactors. Once a set of principles has been established, fibril structure prediction will provide a critical test of our understanding. State-of-the-art protein structure prediction tools such as AlphaFold [59] are currently unable to recapitulate observed fibril structures or their polymorphs, indicating that multiple sequence alignments are inappropriate for finding structures that have not been shaped by evolution, and the complexity of amyloid assembly energy landscapes will require different approaches to navigate computationally. Nonetheless, machine learning is promising for amyloid structure prediction [48], and models trained on deep mutational scanning datasets can already accurately predict sequence regions that stabilize the amyloid core [46] or facilitate conversion to the fibril state [60]. Despite this progress, fibril fold prediction remains in its infancy and accurate predictions of polymorphism will likely require approaches that are able to navigate extensive conformational landscapes with many comparable minima. This will benefit from an improved understanding of how sequence regions pair to build fibril folds [9], as well as targeted strategies to account for kinetic considerations, physiological conditions, and cofactor binding.

There is also an urgent need to better understand the dynamic mechanisms that shape amyloid polymorphism *in vivo*, such as thermodynamic selection, kinetic effects and degradation by chaperones or proteases, and how these relate to cell-type vulnerability in different disease states. This will require high-resolution *ex vivo* structural data to be integrated with high-throughput techniques that are able to rapidly, accurately, and interpretably detect differences in polymorphism, and can be used in kinetic or mechanistic studies where the time taken for exhaustive structure determination would be prohibitive. Promising approaches include computational characterization of fibril morphology in EM or atomic force microscopy (AFM) images [33] and characterization using panels of polymorph-sensitive fluorescent dyes [61]. In addition, biophysical and computational analyses will be needed to elucidate the thermodynamic drivers and mechanistic basis of these processes, and *in situ* fibril structure determination will provide a powerful link between mechanisms and pathology [62]. A better understanding of the mechanisms that shape the evolution of amyloid fibril structure will be crucial to explain how toxicity occurs in these devastating protein folding diseases and to inspire the next generation of amyloid biomarkers and therapeutic strategies.
